# Simulating soil salinity dynamics, cotton yield and evapotranspiration under drip irrigation by ensemble machine learning

**DOI:** 10.3389/fpls.2023.1143462

**Published:** 2023-06-07

**Authors:** Zewei Jiang, Shihong Yang, Shide Dong, Qingqing Pang, Pete Smith, Mohamed Abdalla, Jie Zhang, Guangmei Wang, Yi Xu

**Affiliations:** ^1^ College of Agricultural Science and Engineering, Hohai University, Nanjing, China; ^2^ State Key Laboratory of Hydrology-Water Resources and Hydraulic Engineering, Hohai University, Nanjing, China; ^3^ Cooperative Innovation Center for Water Safety & Hydro Science, Hohai University, Nanjing, China; ^4^ CAS Key Laboratory of Coastal Environmental Processes and Ecological Remediation, Yantai Institute of Coastal Zone Research (YIC), Chinese Academy of Sciences (CAS), Shandong Key Laboratory of Coastal Environmental Processes, YICCAS, Yantai, Shandong, China; ^5^ Shandong Saline-Alkali Land Modern Agriculture Company, Dongying, China; ^6^ Nanjing Institute of Environmental Sciences, Ministry of Ecology and Environment, Nanjing, China; ^7^ Institute of Biological & Environmental Sciences, University of Aberdeen, Aberdeen, United Kingdom

**Keywords:** salinity, evapotranspiration, drip irrigation, cotton, ensemble machine learning

## Abstract

Cotton is widely used in textile, decoration, and industry, but it is also threatened by soil salinization. Drip irrigation plays an important role in improving water and fertilization utilization efficiency and ensuring crop production in arid areas. Accurate prediction of soil salinity and crop evapotranspiration under drip irrigation is essential to guide water management practices in arid and saline areas. However, traditional hydrological models such as Hydrus require more variety of input parameters and user expertise, which limits its application in practice, and machine learning (ML) provides a potential alternative. Based on a global dataset collected from 134 pieces of literature, we proposed a method to comprehensively simulate soil salinity, evapotranspiration (ET) and cotton yield. Results showed that it was recommended to predict soil salinity, crop evapotranspiration and cotton yield based on soil data (bulk density), meteorological factors, irrigation data and other data. Among them, meteorological factors include annual average temperature, total precipitation, year. Irrigation data include salinity in irrigation water, soil matric potential and irrigation water volume, while other data include soil depth, distance from dripper, days after sowing (for EC and soil salinity), fertilization rate (for yield and ET). The accuracy of the model has reached a satisfactory level, *R^2^* in 0.78-0.99. The performance of stacking ensemble ML was better than that of a single model, i.e., gradient boosting decision tree (GBDT); random forest (RF); extreme gradient boosting regression (XGBR), with *R^2^* increased by 0.02%-19.31%. In all input combinations, other data have a greater impact on the model accuracy, while the *RMSE* of the S1 scenario (input without meteorological factors) without meteorological data has little difference, which is -34.22%~19.20% higher than that of full input. Given the wide application of drip irrigation in cotton, we recommend the application of ensemble ML to predict soil salinity and crop evapotranspiration, thus serving as the basis for adjusting the irrigation schedule.

## 1 Introduction

Cotton (Gossypium barbadense L.) is a crucial economic crop in the world (Rodriguez-Sanchez et al., 2022). It is not only one of the main sources of natural fibers for textiles as well as edible oil (Ibrahim et al., 2022), but also plays an important role in national defense, medicine, the automobile industry, and other fields (Xu et al., 2021). Meanwhile, more than 1 billion hectares of soil in the world are threatened by soil salinization, thus land degradation, food reduction (even up to 50%), and environmental threats are increasing day by day (Wang et al., 2021). In arid or semi-arid areas where cotton is widely planted, more than half of the irrigation systems are related to salinization (Wang et al., 2018). Under the background of human growth and global warming, water resources are becoming increasingly scarce, and the traditional irrigation-drainage balance to wash salt method is difficult to maintain, especially in cotton-producing areas such as the arid areas in northwest China (Zong et al., 2022). Smart irrigation based on Internet to Things technology has also paved the way for developing precision irrigation technologies (Boursianis et al., 2021). Thus, drip irrigation, a more water-saving and efficient irrigation technology, has been widely promoted in cotton planting. The way salt moves and accumulates in the soil may be affected by poor drainage, irrigation practices, vegetation removal, and landscape remodelling through earthworks (Sun et al., 2012). Therefore, the dynamics of soil salt in cotton fields under drip irrigation is a problem worthy of study, because the accurate prediction of soil salinity is the most prominent and economic method to prevent soil salinization (Xiao et al., 2023).

Soil salinity is generally expressed by the percentage of salt content (Vermeulen and Van Niekerk, 2017), or electrical conductivity (EC) detected by electromagnetic induction or dielectric sensor. Conventional soil salinity measurement methods include direct assay in the laboratory through chemical methods (Peng et al., 2016), as well as indirect methods such as remote sensing inversion and soil reflectance conversion (Zhao et al., 2022). However, field measurement requires destructive sampling of soil, which is also time-consuming, laborious, and expensive. Electromagnetic induction (EMI), such as EM38 or EM31, can be used as mature methods and auxiliary data to quickly map soil properties related to salinity and measure apparent EC as well (Castrignanò et al., 2012; Narjary et al., 2019). Although the method of satellite remote sensing can be used to identify soil salinity in a large area, its accuracy still needs to be improved, and it is mainly concentrated in the surface soil. Previous studies (Yang et al., 2020) have shown that the increase of soil salinity doesn’t affect crop production until it exceeds a certain threshold level. However, once the salinity threshold is exceeded, the cotton yield almost linearly decreases with the increase of soil salinity (Oster, 1994). At the same time, accurate prediction of cotton yield is of great significance for coping with climate change, cotton breeding (Ashapure et al., 2020), farmers and stakeholders to make wise decisions, such as water and fertilizer input, storage demand, cash flow calculation, crop insurance, etc (Xu et al., 2021). Similarly, the traditional yield measurement methods are either large-scale harvesting in the harvest season, destructive sampling, or remote sensing estimation. However, the former is too time-consuming, while the latter is difficult to improve the resolution and is vulnerable to weather problems such as clouds. In addition, evapotranspiration (ET) of cotton, the loss of water vapor flux transmitted from land and vegetation to air, represents the productivity of crops and is an important indicator for studying the relationship between crop yield and water content (Bhattacharya et al., 2011). ET also plays a key role in the water, carbon and energy cycle of terrestrial ecosystems (Zhang et al., 2022). ET not only affects cotton growth and development, but also influences atmospheric circulation and climate (Huang et al., 2019). The commonly used reference ET (ET_0_) method requires a large amount of meteorological data input, which is difficult to achieve in remote areas (Mattar, 2018). However, the method of crop conversion coefficient (K_c_) depends too much on the accuracy of K_c_, with great uncertainty. To sum up, accurate and practical alternative prediction methods are urgently needed for soil salinity, EC, cotton yield, and ET of drip-irrigated cotton fields. Moreover, considering the advantages of traditional process-based models and data-driven models that can overcome the problem of time-consuming and costly fields trails (Jiang et al., 2023a; Jiang et al., 2023b), this provides a new choice for the prediction of those issues.

A common method is to use hydrological models, such as Hydrus-2D and DRAINMOD, to simulate the water, salt and heat transport process of cotton field system under drip irrigation (Li et al., 2018; Ning et al., 2021). They can be used as a powerful complement to experiments to assess soil hydraulic properties, boundary conditions, irrigation frequency and salinity, and crop types to optimize soil and water management practices (Devkota et al., 2022). However, they require more input data and higher user skills (**Table S1**), which may be difficult to obtain in many regions where data are scarce ad undeveloped. Although widely proven that hydrological models are more interpretable (Liu et al., 2021), they are not always easy to use models and are mainly concentrated on smaller spatiotemporal scales. Most Hydrus studies focused on a depth of 2 m and a range of one year. In addition, previous studies (Karandish and Šimůnek, 2016; Elnesr and Alazba, 2017) have compared hydrological models with ML and found that the former is very sensitive to boundary and initial conditions. If excessive relaxation occurs, the hydrological model may be unstable and can have difficulties in term of speed and convergence probability. Considering that accuracy is proportional to the hardware resources required, it require a higher level of human skills than data-driven models such as ML. Another potential alternative might be machine learning (ML), which is different from the limited regional empirical model and is not as complex and demanding as the hydrological model (Kisi, 2016). Being good at solving nonlinear and multivariable problems, ML has been widely used in hydrology, agriculture, environment, and other fields in recent years (Wan and Goudos, 2020; Jiang et al., 2022a). Among them, ML has been proved to be a powerful tool in crop ET and yield prediction (Dramsch, 2020; Filippi et al., 2022). However, although ML has been used for soil salinity prediction in remote sensing and other fields, it is more concentrated in reflectance and other aspects. Dynamic prediction of soil salinity or EC in drip-irrigated cotton fields based on ML has not been reported. In addition, cotton yield includes not only seed yield, but also lint yield, one of the most important criteria for selecting new lines in breeding (Rodriguez-Sanchez et al., 2022). Hence, we assume that soil salinity, EC, seed yield, lint yield and ET of cotton field under drip irrigation can be predicted by ML and simple input parameters.

Moreover, to compare the effects of different ML models, a new stacking ensemble ML algorithm was also introduced in this study. Since it integrates the basic ML model, it is usually found to have better prediction performance (Jiang et al., 2022b). Meanwhile, the input parameters of the model have a great impact on the results, so it is necessary to find the most suitable and convenient input combination for ML algorithm. The objectives of this research are: (1) to build a global data set of drip irrigated cotton fields, and verify the feasibility of using ML models to predict soil salinity, EC, cotton yield and ET based on basic soil data, meteorological data, irrigation data, and other data; (2) to compare the performance of three common ML models (gradient boosting decision tree, GBDT; random forest, RF; extreme gradient boosting regression, XGBR) and stacking ensemble ML algorithm; (3) to analyze the influence of different input combinations on the accuracy and stability of models.

## 2 Materials and methods

### 2.1 Machine learning models

In this study, three basic ML models, gradient boosting decision tree (*GBDT*), random forest (*RF*), extreme gradient boosting regression (*XGBR*), and a stacking ensemble ML algorithm were selected. This is because they represent three classical commonly used models, and previous studies (Liu et al., 2020; Jiang et al., 2022c) have found that they have good performances in solving regression problems. Initially we intended to include other basic ML models such as support vector machine (SVM) and multiple perceptions (MLP, a type of neural network-based ML, similar to ANN and DNN), but the preliminary results showed that their performance was significantly worse than those tree-based models, and therefore, decided not to include them in this study. More details can be found in the **Supplementary Materials**. All the codes were implemented in python 3.8 by applying the sklearn (https://scikit-learn.org/) and XGB (https://xgboost.readthedocs.io/en/latest/index.html) packages. The models were run on a laptop equipped with Intel core i5-8300H CPU and NAVID GTX 1050 GPU.

#### 2.1.1 GBDT, RF, XGBR

The GBDT model proposed by (Friedman, 2002) is a widely used method using decision stumps or regression trees as basic learners to solve classification or regression problems. It constructs additive regression models by using the least squares method to sequentially plus a simple parameterized function to the current pseudo residuals, the gradient of the loss functional being minimized, in each iteration. The RF model, based on the theory of random partition selection and random subspace, is a simple bagging ensemble of tree predictors, and the results of each tree were weighted and averaged to achieve the final output (Breiman, 2001). The generalization error converges to the limit with the number of trees increasing and the exchange or change of covariables. Both GBDT and RF combine weak learners, but the difference is that the tree of the former is fitted on the residual of the previous tree, so the biases can be reduced, while the latter reduce the variance (Fan et al., 2018). The RF is obtained by training N decision trees on the training-testing set of N samples by putting back samples. The current approximation of GBDT and the margin function of RF are as Eqs. (1-2) respectively. As supposed by (Chen and Guestrin, 2016), the XGBR based on the lifting method, has integrated all the predictions of weak learners (classification and regression trees) through boosting and additive training strategies. It has been found that XGBR improves the objective optimization function by optimizing the loss function and complexity penalty, thereby preventing overfitting. Moreover, the functions in the XGBR model will automatically perform parallel computing in the training period to reduce calculation costs (Eq. (3)). The regularized objectives were minimized according to Eqs. (4-5).


(1) $$F_{m}(x)=F_{m-1}(x)+\nu \;\gamma_{lm}l(x\in R_{lm})$$

(2) $$mg(X\text{, }Y)=av_{k}I(h_{k}(X)=Y)-\underset{j\neq Y}{\max}av_{k}I(h_{k}(X)=j)$$

(3) $$y_{i}=\phi (x_{\text{i}})=\sum_{k=1}^{K}f_{k}(x_{i})\text{, }f_{k}\in F$$

(4) $$L(\phi )=\sum_{i}l(y_{i}^{\prime },y_{i})+\sum_{k}\Omega (f_{k})$$

(5) $$where\;\Omega (f)=\gamma T+\frac{\lambda }{2}{\left\|w\right\|}^{2}$$

where *F_m_* (*x*) is the function maps x to y, which are jointly fit to the training data in a forward “stage wise” manner to boost approximates. ν is the shrinkage parameter that controls the learning rate, and the smaller values represent better generalization error. *γ_lm_* is a constant value predicted by the decision tree in the region *R_lm_*. *l* is the node of the tree. mg (X, Y) is the margin function of X and Y, representing the extent to which the average number of votes of one tree exceeds the average number of votes of any other class. *I* () is the indicator function. A larger marginal value indicates the degree of confidence in classification or regression. $F=\left\{f(x)=w_{q(x)}\right\}(q:R^{m}\to T,w\in R^{T})$ is the space of regression trees. *q*, *w*, and *T* are the independent tree structure (decision rules), leaf weight corresponding to each *f_k_*, and the number of tree leaves. *l*, *y_i_´*, *yi* and Ω represent differentiable convex loss function, prediction, target, and the regression model function, respectively.

#### 2.1.2 Stacking ensemble model

The stacking or stacked generalization was proposed by (Wolpert, 1992). It constructs multi-level classifiers or regressors hierarchically, and uses the mutual complementarities between basic models to enhance the generalization ability. Based on the leave-one-out cross validation method, the stacking model uses the meta learner to optimally combine the prediction results of the basic models to obtain the final output, while the output of the basic learners will not be trained to avoid overfitting. More information can be found in previous studies (Chou et al., 2014; Gu et al., 2022). The weights of base learners were calculated as Eq. (6). In addition, the objective function was computed as follows [Eq. (7)] to estimate stacking weights by minimizing the mean square linear regression.


(6) $$y_{p,i}=\sum_{m=1}^{M}\omega_{m}f_{m,i}$$

(7) $$\Omega =\text{argmin}\sum_{i=1}^{N}{\left(y_{o,i}-\sum_{m=1}^{M}\omega_{m}f_{m,i}\right)}^{2},\text{ (}\sum_{m=1}^{M}\omega_{m}=1,\omega_{m}\geq 0)$$

where *ω_m_* is the weight assigned to each base model, *f_m,i_* represents the prediction of the *i_th_* observation by model m. Ω = {*ω_1_*, *ω_2_*, …, *ω_m_*} denotes the weight set assigned to the base model.

### 2.2 Model simulation and evaluation

#### 2.2.1 Data collection

**Figure 1:**
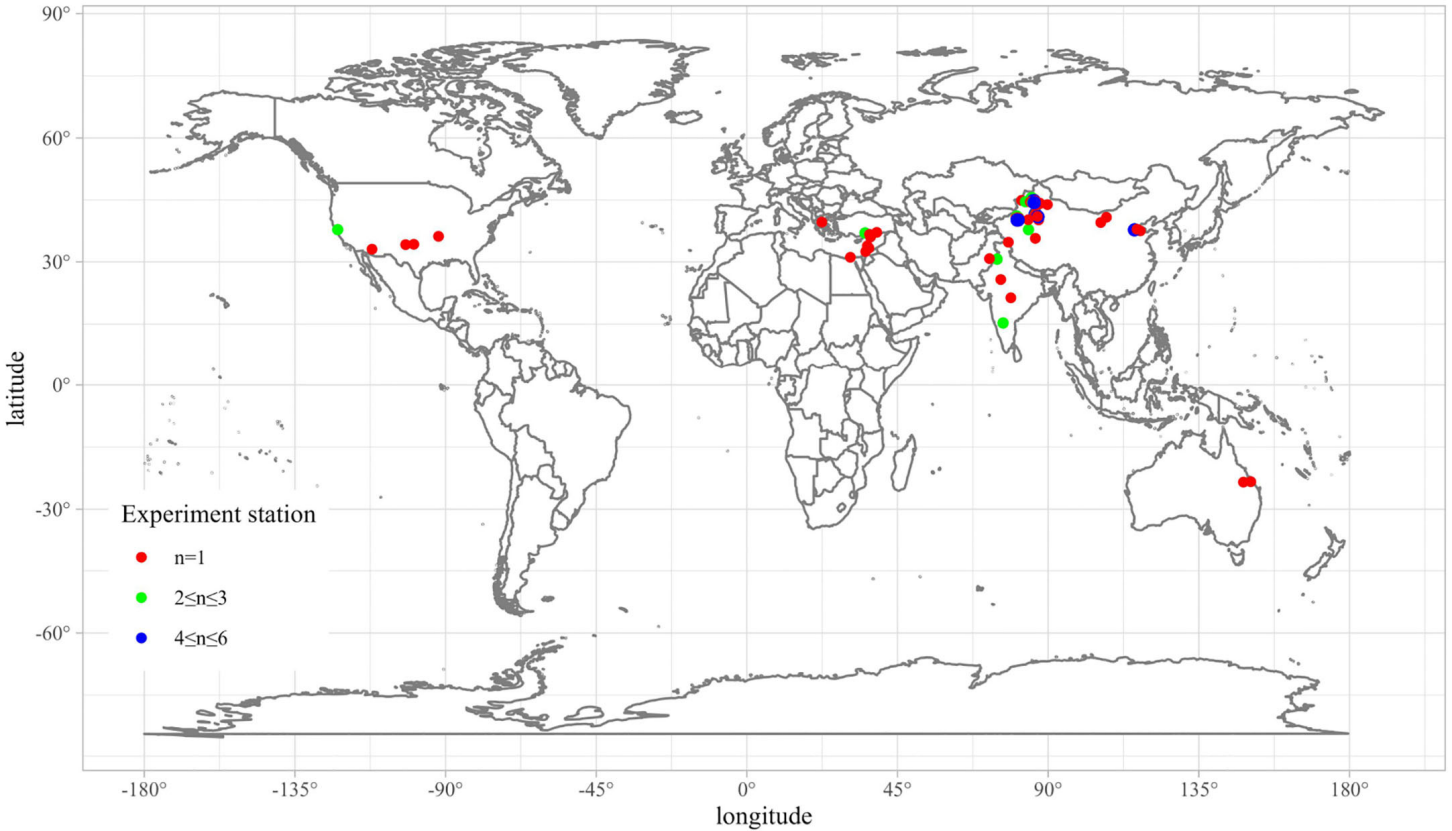
Location of experiment station used in this study. n represents the number of literatures, n > 1 represents multiple trials conducted at the same site.

To obtain the global dataset of soil salinity, cotton yield and ET from cotton fields under drip irrigation, we conducted a comprehensive literature search on the Web of Science and the China Knowledge Resource Integrated (CNKI) database before October 2022. The following keywords were used: soil salt, soil salinity, drip irrigation, evapotranspiration, cotton, and yield. The complete search format was available in the **Supplementary Materials**. All the literature were downloaded and checked manually to ensure they are point measurements, follow a standard data collection protocol, and weather data are from the local or nearest meteorological station. Since it’s hard to make sure all of the drip irrigation setups from global experiments are the same, we used the distance from drip to partly represent line spacing. More details of the uncertainty could be found in the discussion. The total number of papers is 1317, but only 230 papers (50 about ET, 112 about salt, and 68 about yield) were selected after excluding greenhouse, pot, laboratory, and modelling research. Then the abstracts were reviewed carefully to check whether they meet the inclusion criteria, only field trials with multiple replicate samples and details about the experiments. Finally, we collected 134 pieces of literature that are suitable for this study (**Supplementary Materials**). As shown in **Figure 1**, the experiment sites of cotton under drip irrigation cover several continents including Asia, Europe, America, Oceania, and Africa. Data source is from peer-reviewed literatures from database. Data type are xlsx or csv data which could be found in the revised **Supplementary Materials** now. Data structure is matrixes of 10 dimensions, 1514 columns (10, 1514) and (10, 1748) for EC and soil salinity, and matrixes of (8, 232), (8, 121), and (8, 312) for ET, lint yield and grain yield, respectively. The output is a single column vector, and the sample size is the same as the number of the input rows. Sensor type is float for most data except integer for year. Spatial and temporal resolution are globally and yearly for yield and ET (but the salinity includes some daily results). Data size has been mentioned before. Public availability is open access now.

#### 2.2.2 Model inputs, outputs and K-fold cross-validation

On the basis of referring a previous study (Xiao et al., 2023), four types of data were selected for model inputs, soil data (bulk density), meteorological factors (average temperature, total precipitation, year), irrigation data (salinity in irrigation water (SIW), soil matric potential (SMP), irrigation) and other data (soil depth, distance from dripper, days after sowing (DAS) for EC and soil salinity, fertilization for yield and ET). The dataset was divided into several sub datasets since the model inputs available are not the same in different literature, and the outputs are soil EC, salinity, seed yield, lint yield and ET respectively (**Table 1**). Given the economic attributes of cotton, data of seed yield and lint yield were collected. In addition, due to the different emphasis of different studies on soil salinity, we have collected data on soil EC and salt content. This was because there is a positive correlation between them, but they cannot be accurately transformed now (Phonphan et al., 2014). It is obviously soil data and meteorological factors will influence the results; the irrigation data was selected to estimate the effects of drip irrigation. Soil salinity and EC were related to soil depth, dripper position and cotton growth stage. Cotton yield was significantly related to fertilization, which also affected crop growth and ET. Soil data, meteorological factors and irrigation data were mandatory, while the other data was different for salinity, yield and ET. The detailed model inputs of different outputs are also available in **Table 2**. The dataset was randomly split to the training and testing sets by a commonly used ratio of 70%:30% according to a previous study (Livera et al., 2019). Specifically, the sample sizes of training-testing subsets of EC, soil salinity, seed yield, lint yield, and ET are 1060 and 454, 1224 and 524, 261 and 112, 85 and 37, 162 and 70 respectively. Moreover, 10-fold cross-validation was conducted to avoid over-fitting and the hyperparameters were optimized by grid search and trial and error (**Table S2**).

**Table 1:** Collected global dataset.

	Bulk density	SIW	SMP	Temp	Prec	Irrigation	Soil depth	Distance	Year	DAS	Fertilization	EC	Soil salinity	Seed yield	Lint yield	ET
Units	g cm^-3^	g L^-1^	-kPa	°C	mm	mm	cm	cm	y	d	kg N ha^-1^	mS cm^-1^	g kg^-1^	kg ha^-1^	kg ha^-1^	mm
n	3987	3987	3987	3987	3987	3987	3262	3262	3987	3262	725	1514	1748	372	121	232
mean	1.49	1.75	5.74	10.69	160.10	466.60	60.67	6.33	3.26	118.42	228.08	2.00	7.17	5117.42	2470.18	571.94
max	1.75	9.00	50.00	29.40	1168.70	1032.00	650.00	99.00	22.00	360.00	480.00	15.68	46.03	7720.00	3899.00	1129.43
min	1.16	0.20	1.50	5.10	0.00	0.00	0.00	0.00	0.00	0.00	0.00	0.03	0.06	1266.83	1006.00	272.00
STD	0.10	2.15	3.89	3.53	150.80	249.22	52.37	15.60	3.98	96.60	124.00	2.23	7.17	1458.90	695.48	220.07

SIW, SMP, Temp, Prec, distance, DAS and n denote salinity of irrigation water, soil matric potential, average annual temperature, Annual precipitation, distance from dripper, days after sowing and number respectively.

**Table 2:** Input combinations for scenario simulation.

Scenarios	S0	S1	S2
Models	GBDT0, RF0, XGBR0, Stacking0	GBDT1, RF1, XGBR1, Stacking1	GBDT2, RF2, XGBR2, Stacking2
EC/Soil salinity	Bulk density, SIW, SMP, Temp, Prec, Irrigation, Soil depth, Distance, year, DAS	Bulk density, SIW, SMP, Irrigation, Soil depth, Distance, year, DAS	Bulk density, SIW, SMP, Temp, Prec, Irrigation
Seed/lint yield/ET	Bulk density, SIW, SMP, Temp, Prec, Irrigation, year, Fertilization	Bulk density, SIW, SMP, Irrigation, year, Fertilization	Bulk density, SIW, SMP, Temp, Prec, Irrigation

SIW, SMP, Temp, Prec, distance, and DAS denote salinity of irrigation water (g L^-1^), soil matric potential (-kPa), average annual temperature (°), Total precipitation (mm), distance from dripper (cm), and days after sowing (d) respectively.

#### 2.2.3 Scenarios simulation

To test the effects of the four kind of input data (soil data, meteorological data, irrigation data, and other data) on model accuracy, three scenarios (S0, S1, S2) of input combinations were set up for simulation. Among them, S0 is all data as input, S1 represents input without meteorological factors, S2 represents input without other data. The detailed information can be found in **Table 2**.

#### 2.2.4 Evaluation criteria

In this study, we selected three commonly used criteria, i.e., mean absolute error (*MAE*), root mean square error (*RMSE*), and coefficient of determination (*R^2^*) to evaluate the model performance (accuracy and stability) as follows.


(8) $$MAE=\frac{1}{n}\sum_{i=1}^{n}\left|Y_{O,i}-Y_{P,i}\right|$$

(9) $$RMSE=\sqrt{\frac{\sum_{\text{i}=1}^{n}{\left(Y_{O,i}-Y_{P,i}\right)}^{2}}{n}}$$

(10) $$R^{2}=1-\frac{\sum_{i=1}^{n}{\left(Y_{O,i}-Y_{P,i}\right)}^{2}}{\sum_{i=1}^{n}{\left(Y_{O,i}-\bar{Y_{O}}\right)}^{2}}$$

where *Y_O,i_*, *Y_P,i_*, $\bar{Y_{O}}$ and n represent the observed, predicted, and mean of observed values (soil EC, salinity, seed yield, lint yield and ET). The closer *R^2^* is to 1, the closer *RMSE* and *MAE* are to 0, and the higher the accuracy of the model.

## 3 Results

### 3.1 Overview of the global datasets and the relationship among inputs and outputs

**Figure 2:**
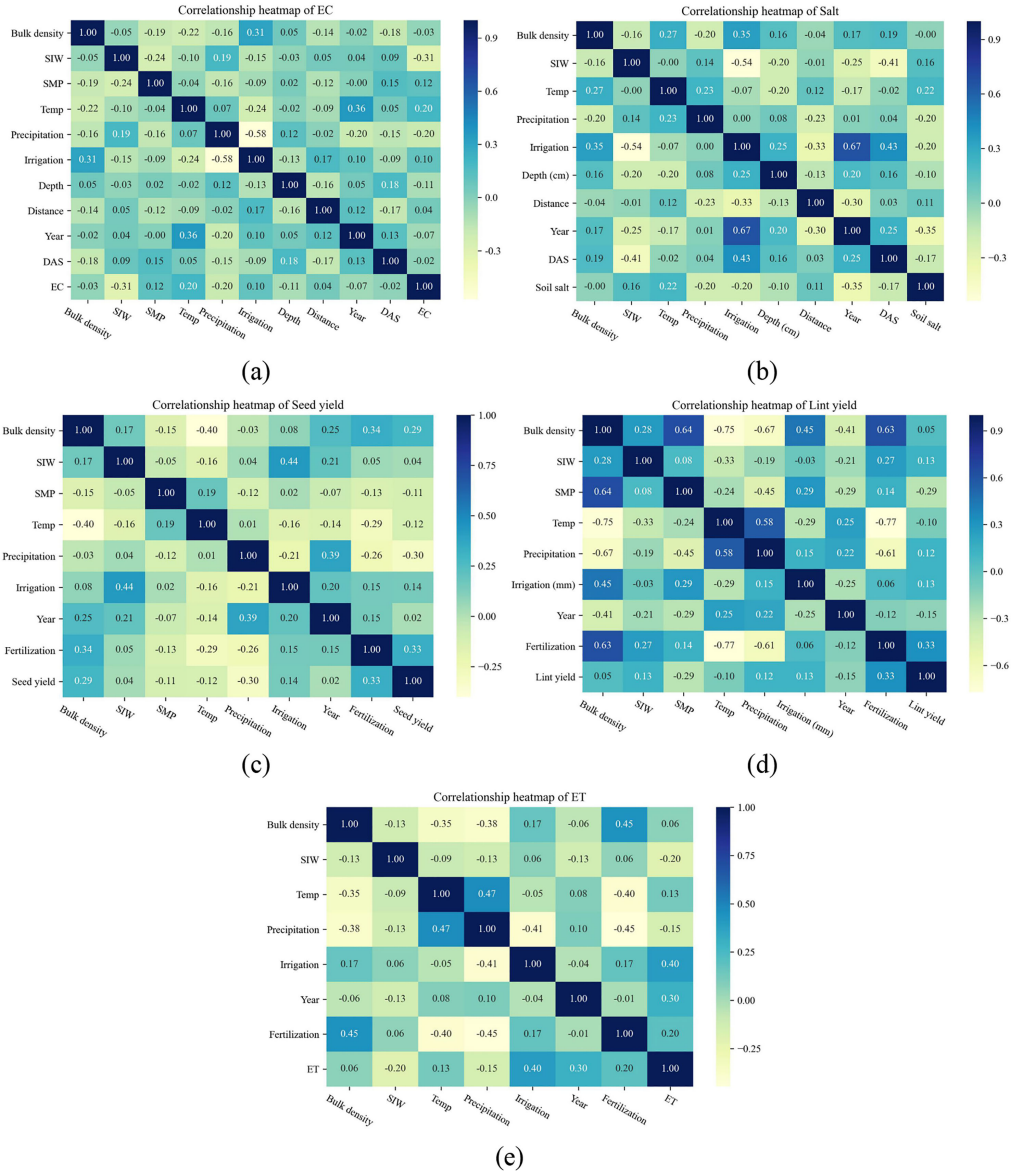
Spearman correlation heatmap of input parameters and conductivity (*EC*, **A**), soil salt **(B)**, seed yield **(C)**, lint yield **(D)**, and *ET* **(E)**. The *SIW*, *SMP*, *Temp*, *DAS* are salinity of irrigation water (g L^-^¹), soil matric potential (kPa), temperature (°C), and days after sowing respectively. The units of bulk density, precipitation, irrigation, and fertilization are g cm^-^³, mm, mm, kg N ha^-1^. The units of *EC*, soil salt, seed yield, lint yield, and *ET* are mS cm^-1^, g kg^-1^, kg ha^-1^, kg ha^-1^, and mm, respectively.

The overview of the global dataset obtained in this study can be found in **Table 1**. In general, the dataset includes five sub data sets, soil EC, soil salinity, seed yield, lint yield, and ET, and their sample sizes are 1514, 1748, 373, 121, and 232, respectively. To explore the relationship between model inputs and prediction objectives, and verify the feasibility of our selection of input parameters, we built the Spearman correlation heatmap between inputs and outputs (**Figure 2**). SMP was positively correlated with EC and salt. However, it is worth pointing out that the negative correlation between SIW and EC is not reliable, because the SIW research mainly focused on soil salinity, and most of the EC studies have no SIW description (mostly -5 kPa). It can be easily found that SIW, temperature, and distance from drippers were positively related to soil salinity (**Figure 2B**), while precipitation, irrigation, depth, year, and DAS were negatively correlated with salinity. This is understandable because the greater the salinity of the irrigation water, the farther away from the emitter, the less the soil was washed, and the greater the possibility of salt residue. Irrigation could wash away soil salt, and with the growth of cotton (DAS and year increasing), the soil salinity will reduce, which was consistent with previous studies (Phogat et al., 2012; Wang et al., 2018). When it comes to the relationship heatmap of cotton yield (**Figure 2C, D**), there was a positive correlation between fertilization, irrigation, soil bulk density and yield, no matter seed yield or lint yield, while a negative correlation was found between SMP, temperature and yield. The positive correlation between cotton yield and fertilization amount in a certain range has been confirmed by many studies (Dong et al., 2010; Ibrahim et al., 2022). The negative correlation between SMP and yield may be explained by the inhibition of salt and high temperature. As for the positive correlation between ET and irrigation, year, fertilization, and the negative correlation with rainfall (**Figure 2E**), it may be explained by the change of seed cotton yield (**Figure 2C**).

### 3.2 Performance of ML models modelling EC and soil salinity

**Figure 3:**
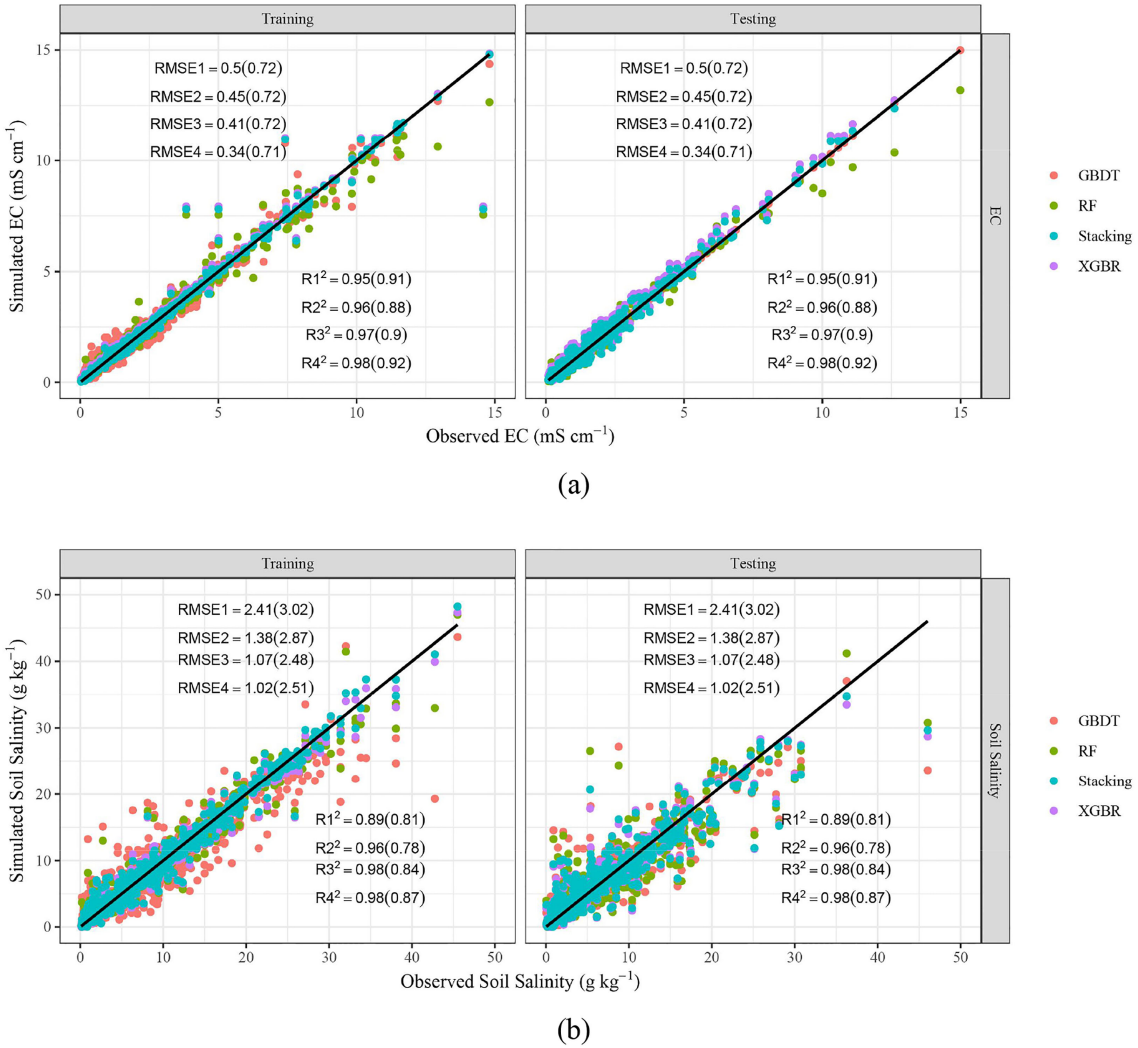
Scatter plots of predicted and observed EC **(A)** and Soil salinity **(B)**. *R_1_^2^*, *R_2_^2^*, *R_3_^2^*, *R_4_^2^*, *RMSE_1_*, *RMSE_2_*, *RMSE_3_*, *RMSE_4_* represent *R²* and RMSE (unit: mS cm^-1^, a, g kg^-1^, b) of GBDT, RF, XGBR, Stacking respectively. The values outside and within brackets represent the results of the training and testing periods.

**Figure 3** show the results of EC and soil salinity simulated by different ML models (GBDT, RF, XGBR, and stacking). It could be found that most of the points are very close to the 1:1 line, which denotes that the ML algorithms could predict EC and soil salinity. As illustrated by **Table 3**, three basic models (GBDT, RF, XGBR) could capture the dynamics of both EC and soil salinity, with *R^2^* ranging from 0.89 to 0.98 and 0.78 to 0.91 during the training and testing periods respectively. While *MAE* of EC and soil salinity were 0.05-0.14 mS cm^-1^ and 0.63-1.49 g kg^-1^ (training), 0.27-0.34 mS cm^-1^ and 1.56-1.89 g kg^-1^ (testing). By contrast, the stacking ensemble ML model performed the best, with *R^2^*, *MAE*, *RMSE* of EC and soil salinity in 0.92-0.98 and 0.87-0.98, 0.05-0.27 mS cm^-1^ and 0.61-1.55 g kg^-1^, 0.34-0.71 mS cm^-1^ and 1.02-2.48 g kg^-1^. During the training stage, the *R^2^* of stacking model increased by 1.10%-2.65% (EC) and 0.34%-10.07% (soil salinity), with *MAE* and *RMSE* decreased by 40.86%-62.19% and 3.62%-59.44% (EC), 18.68%-33.06% and 4.95-57.79% (soil salinity), respectively, compared with basic models. While in the testing period, the corresponding *R^2^* increased by 1.68%-4.64% (EC) and 3.20%-10.52% (salinity), *MAE* and *RMSE* decreased by 0.40%-19.22% and 0.09%-1.37% (EC), 0.47%-18.02% and 1.20%-17.82% (salinity), respectively. In addition, the calculating time of XGBR and stacking model was almost the smallest among these three models, especially during the testing stage.

**Table 3:** Performance of four ML models for predicting EC and soil salinity during training and testing stages.

Periods	Training				Testing			
Models	GBRT	RF	XGBR	Stacking	GBRT	RF	XGBR	Stacking
EC								
Time (s)	0.20	0.36	0.10	0.10	0.24	0.27	0.07	0.01
R^2^	0.95	0.96	0.97	0.98	0.91	0.88	0.90	0.92
MAE	0.09	0.14	0.10	0.05	0.34	0.27	0.30	0.27
RMSE	0.50	0.45	0.41	0.34	0.72	0.72	0.72	0.71
Soil salinity								
Time (s)	0.36	0.29	0.54	0.18	0.24	0.26	0.07	0.01
R^2^	0.89	0.96	0.98	0.98	0.81	0.78	0.84	0.87
MAE	1.49	0.75	0.63	0.61	1.89	1.71	1.56	1.55
RMSE	2.41	1.38	1.07	1.02	3.02	2.87	2.48	2.51

### 3.3 Simulation accuracy of seed yield and lint yield

**Figure 4:**
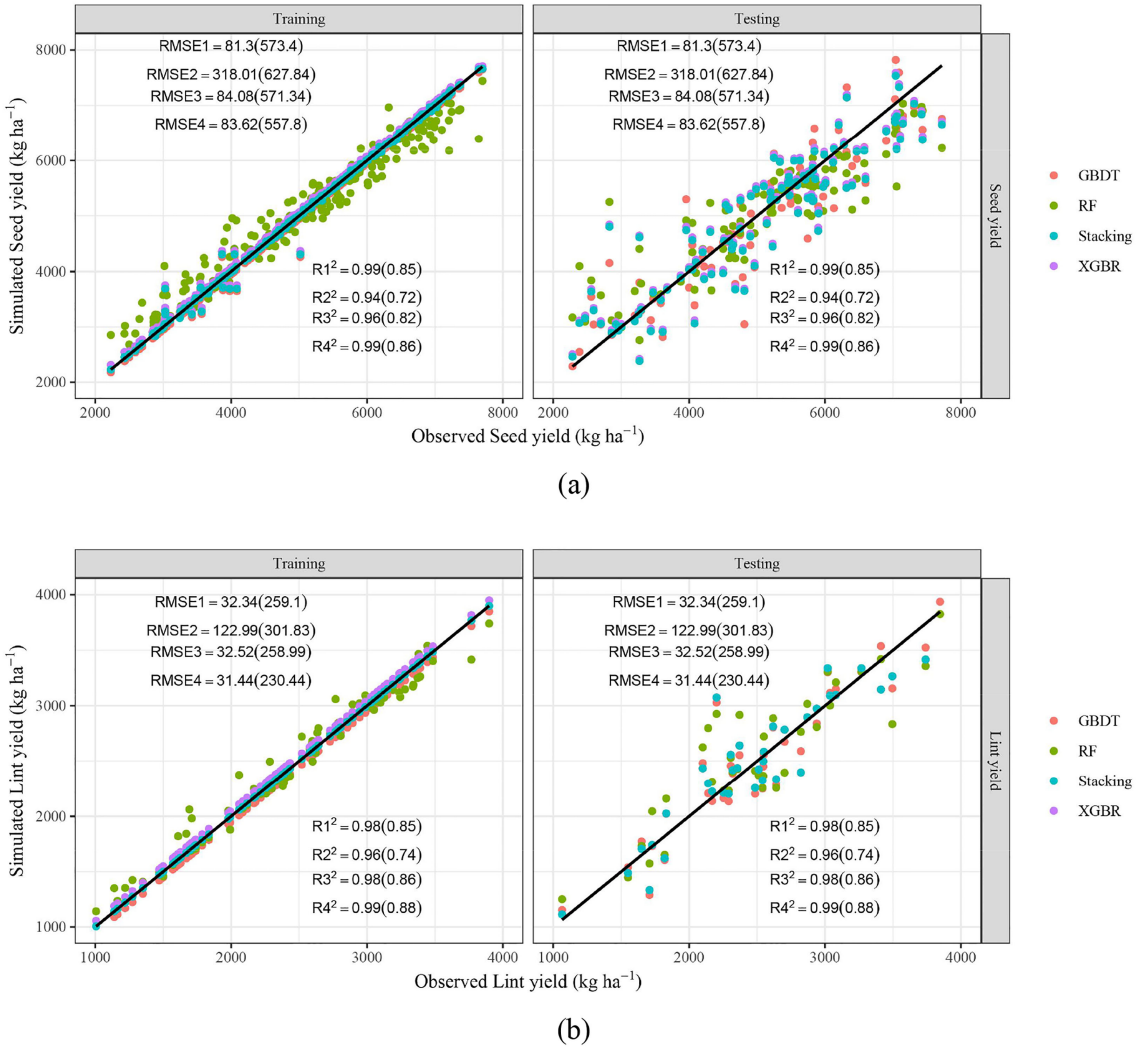
Scatter plots of predicted and observed seed yield **(A)** and lint yield **(B)** of cotton. *R_1_^2^*, *R_2_^2^*, *R_3_^2^*, *R_4_^2^*, *RMSE_1_*, *RMSE_2_*, *RMSE_3_*, *RMSE_4_* represent *R^2^* and *RMSE* (unit: kg ha^-^¹) of GBDT, RF, XGBR, Stacking respectively. The values outside and within brackets represent the results of the training and testing periods.

**Figure 4** offers the scatter plots of seed yield (a) and lint yield (b) predicted by four ML models. It shows that although the performance was worse than soil salinity, the points are still near the 1:1 line, especially the blue points (stacking ensemble model). **Table 4** illustrated the modelling performance of cotton yield by different ML models. Specifically, the *R^2^*, *MAE* and *RMSE* of seed yield and lint yield predicted by three basic models (GBDT, RF, and XGBR) were in 0.94-0.99, 15.63-216.47 kg ha^-1^, 81.30-318.01 kg ha^-1^ and 0.96-0.98, 0.32-92.08 kg ha^-1^, 32.34-122.99 kg ha^-1^ (training stage), 0.72-0.85, 418.08-442.00 kg ha^-1^, 571.34-627.83 kg ha^-1^ and 0.74-0.86, 190.27-231.51 kg ha^-1^, 258.99-301.83 kg ha^-1^ (testing stage), respectively. Similarly, the stacking ensemble ML model also obtained the best performance, with *R^2^*, *MAE*, and *RMSE* in, 0.86-0.99, 28.66-396.61 kg ha^-1^, 83.62-557.80 kg ha^-1^ (seed yield), and 0.88-0.99, 0.13-169.24 kg ha^-1^, 31.44-230.44 kg ha^-1^ (lint yield) respectively. The *R^2^* of seed yield and lint yield were 0.02%-19.31% and 1.02%-18.11% higher than basic models, while those *MAE* and *RMSE* were and 5.14%-92.78% and 2.37%-74.43%, 11.05%-99.86% and 2.77%-74.43% lower, respectively.

**Table 4:** Performance of four ML models for predicting seed yield, lint yield and ET of cotton during training and testing stages.

Periods	Training				Testing			
Models	GBRT	RF	XGBR	Stacking	GBRT	RF	XGBR	Stacking
Seed yield								
Time	0.81	0.14	0.04	0.01	0.83	0.14	0.04	0.01
R^2^	0.99	0.94	0.99	0.99	0.85	0.72	0.82	0.86
MAE	15.63	216.47	30.20	28.86	419.74	442.00	418.08	396.61
RMSE	81.30	318.01	84.08	83.62	573.40	627.83	571.34	557.80
Lint yield								
Time	0.46	0.02	0.03	0.01	0.51	0.02	0.04	0.01
R^2^	0.98	0.96	0.98	0.99	0.85	0.74	0.86	0.88
MAE	0.32	92.08	0.32	0.13	190.36	231.51	190.27	169.24
RMSE	32.34	122.99	32.52	31.44	259.10	301.83	258.99	230.44
ET								
Time	0.63	0.94	0.06	0.01	0.64	0.72	0.04	0.01
R^2^	0.99	0.99	0.99	0.99	0.95	0.87	0.94	0.97
MAE	2.01	16.17	2.01	1.72	32.85	46.07	32.81	30.21
RMSE	6.63	23.02	6.63	6.61	51.75	69.94	51.68	41.47

### 3.4 Comparison of different ML models predicting ET

**Figure 5:**
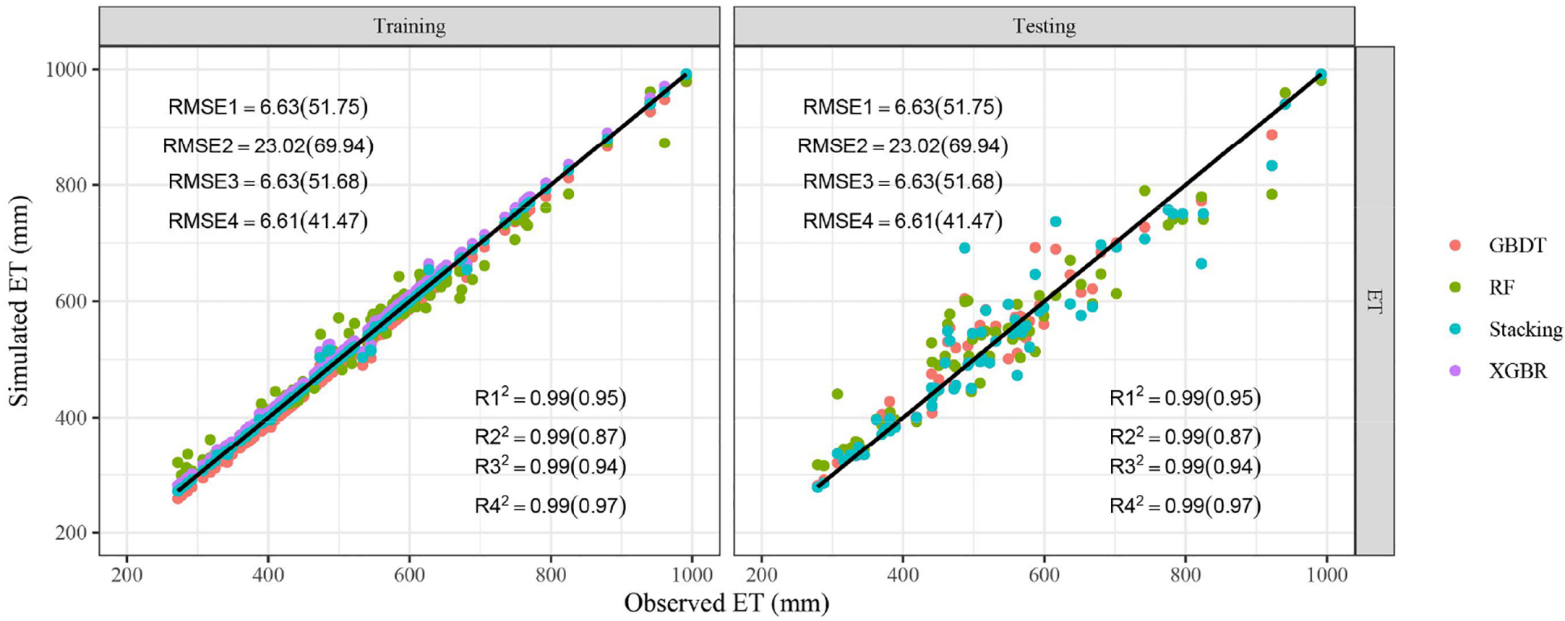
Comparison of predicted and observed ET. *R_1_^2^*, *R_2_^2^*, *R_3_^2^*, *R_4_^2^*, *RMSE_1_*, *RMSE_2_*, *RMSE_3_*, *RMSE_4_* represent *R²* and *RMSE* (unit: mm) of GBDT, RF, XGBR, Stacking respectively. The values outside and within brackets represent the results of the training and testing periods.

The ET data used in this study were the seasonal cumulative ET, which were calculated by the water balance method. Specifically, it is the result of irrigation amount, precipitation, the change of soil water storage minus surface runoff and the downward flux below the crop root zone (**Supplementary Materials**). As displayed in **Figure 5**, the stacking ensemble ML algorithm was the closest to the observed ET points, followed by GBDT, XGBR, and RF. The points were almost all distributed around the 1:1 line, which denotes the acceptable accuracy of models. The comparison of model performance was shown in **Table 4**. It could be found that predicting ET was much easier than other outputs based on the soil data, meteorological data and fertilization, with *R^2^*, *MAE*, *RMSE* in 0.99 and 0.87-0.97, 1.72-16.17 mm and 30.21-46.07 mm, 6.61-23.02 mm and 41.47-69.94 mm during the training and testing periods, respectively. Compared with those three basic models (GBDT, RF, and XGBR), the stacking ensemble model obtained the best model performance also, with *R^2^* increased by 1.15%-11.41%, MAE and RMSE decreased by 7.93%-89.39% and 0.27%-71.29% respectively.

### 3.5 Results of scenario simulation

**Figure 6:**
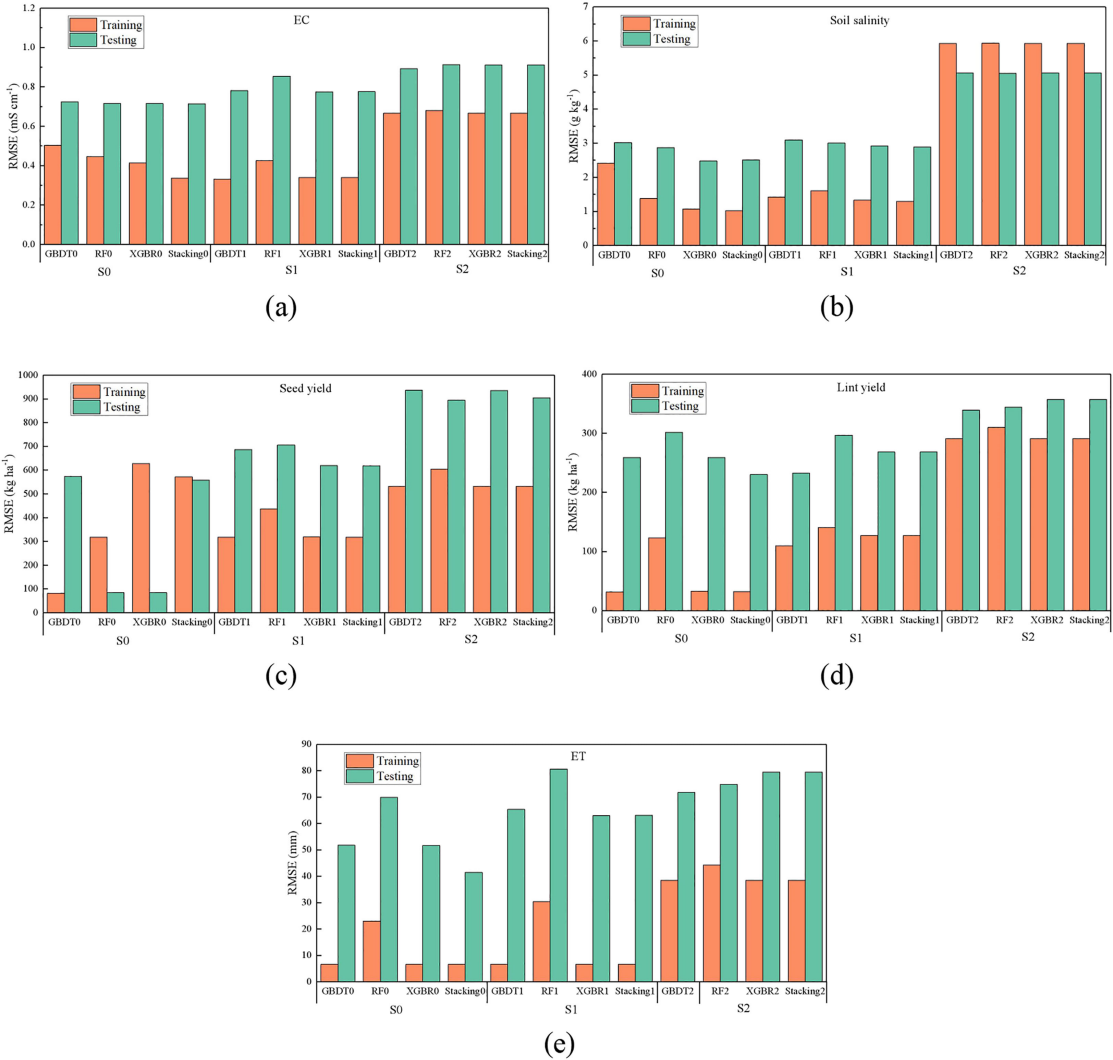
Comparison of RMSE of EC **(A)**, soil salinity **(B)**, seed yield **(C)**, lint yield **(D)**, and ET **(E)** under three scenarios (S1, S2, S3).

To test the impacts of input parameters on model performance, the results of EC, soil salinity, although the *RMSE* of S1 in the training phase was close to or even less than the full inputs scenario, in most cases, especially during the testing period, the *RMSE* of S0 was still the smallest. The RMSE of S1 and S2 in the testing phase increased by 7.80%-19.20% and 23.22%-27.55% than S0, respectively. As seen in **Figure 6C, D**, the model performance with different input combinations varies greatly. In particular, the model accuracy under S2 scenario was significantly reduced. During the testing period, the *RMSE* of yield are 42.32%-63.63% and 26.70%-51.02% (seed), 14.06%-55.05% and 15.99%-46.04% (lint) higher than S0 and S1 respectively. Overall, the *RMSE* of stacking model was the smallest, indicating its best accuracy regardless of input combinations. It could be found that the prediction of ET depends on the meteorological factors and fertilization data also, especially during the testing periods. The *RMSE* of ET predicted by ML models under S0 decreased by 0.29%-52.09% and 6.92%-481.39% than S1 and S2, respectively.

## 4 Discussion

### 4.1 Comparison of different ML models

The soil salinity was affected by soil texture, soil moisture and irrigation, thus those factors should be considered when predicting soil salinity or EC (Wang et al., 2022). Proper evaluation of soil salinity requires traditional laboratory analysis, which is often cumbersome, expensive, time-consuming and laborious (Xiao et al., 2023). The prediction by process-based model requires complex input data and high knowledge, or the spectral reflectance of soil salinization recorded by optical remote sensing satellite (Gorji et al., 2020) or unmanned aerial vehicle (UAV) multispectral data (Zhao et al., 2022). Recently, these methods have also made significant progress, becoming more user-friendly and accurate. More studies (Ivushkin et al., 2017; Ning et al., 2021) have used process-based or remote sensing models to simulate the dynamics of soil salinity in cotton fields, providing guidance for irrigation schedules and water salt balance. What’s more, the leverage of massive data brought by remote sensing has also made ML more widely used. For example, a study (Qi et al., 2022) has proposed a soil salinity monitoring method based on satellite-ground spectral fusion and satellite-UAV collaboration in cotton planting areas, thereby drawing a soil salinity distribution map for cotton fields. In this study, we establish a method to predict soil salinity, EC, seed yield, lint yield and ET based on four ML methods (GBDT, RF, XGBR, stacking ensemble model) and soil data, meteorological data, irrigation data and other data. Overall, the model obtained an acceptable performance especially for the stacking ensemble model. It could be found that both XGBR and GBDT showed a better prediction performance than RF, which was basically consistent with a previous study (El Bilali et al., 2021). In other words, the ability of more complex integration algorithms such as boosting (XGB) and stacking ensemble model to deal with complex problems is significantly higher than that of bagging (RF), which has been proved by previous study (Pham and Won, 2022). The possible reason is that the GBDT is based on the boosting method which establishes a decision tree in each iteration, and the subsequent tree corrects the errors of the previous tree and thereby, continuously approaching the true value (Obsie et al., 2020). This is different from the RF model based on the bagging method which selects training data by putting back the sample and can simultaneously establish multiple independent decision trees (Du et al., 2022). This study mainly focused on tree-based models rather than, among others, SVM, MLP and artificial neural networks. This is because when we test the model performance, tree-based models obtained a much better result than SVM and MLP (**Figure S1**). However, future studies will be critical to investigate this further.

When it comes to the prediction of cotton yield (seed yield and lint yield), the model still performed well, especially the XGB and stacking. Previous studies (Xu et al., 2021; Kaur Dhaliwal et al., 2022) on the importance score of variables for predicting cotton yield by ML found that the yield had the highest response to management variables (nitrogen fertilizer application amount, covering crops, no tillage years), followed by soil and climate variables. This is consistent with our study, which proved the importance of fertilizer, meteorological data and soil bulk density by a global dataset. In addition, the nonlinear relationship among cotton yield and fertilization rate, year has been found (Nouri et al., 2020; Xie et al., 2021). This also partly proves the feasibility of using ML, which is good to solving nonlinear problems, to predict cotton yield. Last but not least, the prediction effect of ET is pretty good, even better than soil salinity and cotton yield. It is understandable since numerous studies (Xu et al., 2018; Zhang et al., 2022) have found that ML is a good tool to predict ET based on meteorological data. Previous studies (Xu et al., 2017) have also found that tree ensemble and boosted regression tree performed well in predicting ET than other ML models, which is in line with this study. The ensemble ML method shows an improvement in the predictability of crop yields, compared to the linear relationship of traditional models, which is consistent with previous studies (e.g., Kaur Dhaliwal et al., 2022). The *R^2^* of process-based models typically ranged from 0.83 to 0.96 (Masasi et al., 2020). This might be because cotton production is affected by many factors, including crop management, soil, and climate parameters. The relationship between yield response and environmental factors are not linear and the process-based models could explain the variance of the linear portion of the response variables better (Xie et al., 2021). Accurate simulation of cotton yield using crop models often requires substantial expertise, intensive data, and extensive calibration compared with ML (Dumont et al., 2015). However, it cannot be ignored that the performance of ML models is limited by the lack of spatial and temporal data covering a wide range of output and prediction variables (Shahhosseini et al., 2019). We suggest that future research combine ML with process-based models to overcome data limitations.

### 4.2 Effects of input combinations on model performance

The performance of ML models is greatly affected by regional climate conditions and the combination of input meteorological factors (He et al., 2020). This study also found this, and not only in predicting ET, but also in soil salinity, EC, seed yield, and lint yield. It is obvious that when all the data, namely soil data, meteorological data, irrigation data and other data, are used as inputs, the accuracy of all ML models is better than that of S1 and S2 scenarios with the combination of some input parameters (**Figure 6**). Actually, it is easily understandable since the movement of soil salt will be affected by management measures such as irrigation, salt washing and meteorological factors (Ren et al., 2019), as well as the location of emitters and soil layers. The cotton yield and ET are obviously be affected by fertilization and meteorological factors (Huang et al., 2019). In addition, many previous studies (Dong et al., 2020; Xiao et al., 2023) have also proved this point. The results of Spearman correlation matrix (**Figure 2**) also showed that there was no significant correlation between most of the input parameters, which also partly supported the necessity of using more comprehensive input parameters to predict soil salinity, cotton yield and ET. As for other data such as soil depth, it was also found (Su et al., 2022) that the migration difficulty of different irons determines their accumulation depth under irrigation and evaporation. When it comes to the difference between S1 and S2, the RMSE of ML models of S1 are lower than S2 in most cases. This partly proved that the importance of other data even rather than meteorological data. It is reasonable when considering the large spatial and temporal variability of soil salinity or EC (soil depth, distance from dripper, and DAS), while cotton yield and ET are obviously affected by crop growth and fertilizer application. Moreover, the prediction accuracy of ET by ML models is higher than soil salinity, which might be because the correlation of ET and input parameters are larger (**Figure 2**). However, it should also be pointed out that the future studies should be critical to find the relationship between input combinations with model performance, since there might be some potential overfitting.

### 4.3 Implications, prospects and limitations

This study proved the ML models, especially the XGBR and stacking ensemble ML algorithm, are useful tools to predict soil salinity, EC, cotton yield and ET. It is encouraging because all the input parameters are not difficult to obtain, the use of the model is relatively simple, and the accuracy and stability are satisfactory. Based on this study, we are confident that we can realize the real-time prediction of soil salinity, ET and cotton yield under drip irrigation in the future. This can be used to guide practice and agricultural production, and optimize the management measures. The model obtained a satisfactory performance overall, even better than Hydrus, for example, previous studies (Hu et al., 2017; Liu et al., 2021) have found *R^2^* in simulating soil salinity ranged from 0.53-0.98, which was smaller than this study. However, there are some factors limit and degrade the current research, which can be further studied. Firstly, the currently predicted soil salinity and EC dynamics are still on a relatively coarse temporal and spatial scale, that is, based on DAS, soil depth, distance from emitters, etc., and whether the model has a more refined (hourly scale) and wider range prediction capability has not been confirmed. Although the distance from drippers reflects some spatial variability, different drip irrigation settings also bring some uncertainty. Secondly, as the types of data input are still limited, whether the model accuracy can be further improved remains to be discussed. This is mainly because the basic parameters measured in different studies are difficult to be completely consistent. The present selection of input parameters takes more into account the availability of data and their relevance to the results. This makes it easier for the model to obtain the input data in actual use, but it may also bring some uncertainties. For example, the temperature and precipitation used in this study are the annual average temperature and cumulative precipitation respectively. In the arid areas where the cotton is planted, the rainfall is very small. However, to make more accurate predictions, it may be better to select the precipitation and temperature during the growth period. But these data are usually lacking in many pieces of literature. In addition, it would be more encouraging to get the dynamic prediction of soil salinity, EC and ET with time series. Although the current research can also fill the gap for the observation data, it is more about the prediction of a certain state. How to achieve more accurate prediction combined with increasingly powerful and robust deep learning (DL) model may be another direction. Initially, we tested the results of the neural network during model selection but found the simulation results not satisfactory (data not shown). We suggest further future research such as integrating basic DL models to investigate this. Last but not least, ML algorithms are often criticized by black boxes because they can obtain good prediction results, but it is difficult to reveal how they are implemented. We have made some analysis of the spearman correlation between the input and output of the model, but it may be possible to further study it through the interpretive ML algorithm in the future (Jones et al., 2022). Moreover, in recent years, graph convolutional network (GCN), knowledge distillation (KD), edge artificial intelligence algorithms and other technologies have shown good potential in unsupervised learning (Gou et al., 2022; Wu et al., 2023; Zhang et al., 2023), but how to apply these methods to the agricultural field remains to be further studied.

## 5 Conclusions

This study displayed the application of different ML models (GBDT, RF, XGBR, and stacking ensemble) to accurately predict soil salinity, EC, seed yield, lint yield, and ET in drip irrigated cotton fields. Based on the global data set collected from 134 literatures, we verified the feasibility of predicting those outputs based on soil data, meteorological data, irrigation data and other data. SIW, temperature, and distance from drippers were positively related to soil salinity, while precipitation, irrigation, depth, year, and DAS were negatively correlated with salinity. The ML models have achieved satisfactory performance in both training and testing stages, and the accuracy of the models are higher in predicting cotton yield and ET than that of soil salinity. The stacking ensemble ML could improve model performance. Taking the prediction of soil salinity and EC for example, the *R^2^* increased by 1.68%-10.52%, *MAE* and *RMSE* decreased by 0.40%-19.22% and 0.09%-17.82%, respectively. When the input parameters of the model are reduced (especially the other data and meteorological data), the accuracy of the model is significantly reduced. Therefore, under the condition of complete input parameters, it is recommended to apply ML algorithm, especially the stacking ensemble ML, to predict soil salt dynamic, ET and cotton yield in drip irrigated cotton fields in arid regions of the world.

## Data availability statement

The original contributions presented in the study are included in the article/**Supplementary Material**. Further inquiries can be directed to the corresponding author.

## Author contributions

ZJ provided the idea, collected the data, run the model, writing the original draft. SY provided the funding and writing- review and editing. SD and QP conducted part of the field data and validated the draft. PS, MA, and YX helped revised the paper and provided suggestions. JZ provided the methodology and edited the paper. GW provided the funding. All authors contributed to the article and approved the submitted version.

## Supplementary material

Table S1 The original dataset collected in this study.
