# Supplementary material for: Simulating soil salinity dynamics, cotton yield and evapotranspiration under drip irrigation by ensemble machine learning
Source: Front Plant Sci. 2023 Jun 7;14:1143462. doi: 10.3389/fpls.2023.1143462 (PMC10282761; doi:10.3389/fpls.2023.1143462)
Supplement: Supplementary file 1 [file DataSheet_1.docx]

Supplementary Material

Prediction of soil salinity dynamics, cotton yield and evapotranspiration under drip irrigation by ensemble machine learning

Zewei Jiang, Shihong Yang*, Shide Dong, Qingqing Pang, Jie Zhang, Guangmei Wang

*** Correspondence:** Corresponding Author: Shihong Yang ([ysh7731@hhu.edu.cn](mailto:ysh7731@hhu.edu.cn)); Pete Smith (pete.smith@abdn.ac.uk)

# Supplementary Data

A detailed description of the global dataset is available in data.xlsx.

# Supplementary Figures and Tables

Table S1 Comparison of the model inputs of ML and Hydrus

| Types | ML | Hydrus |
| --- | --- | --- |
| Weather | Temperature, precipitation | Radiation, precipitation, temperature, moisture (optional) |
| Soil geometry information | Soil depth | Number of soil materials, number of layers for mass balance, decline from vertical axes, depth of the soil profile |
| Soil hydraulic parameters | Bulk density | *θ_r_*, *θ_s_*, *α*, *n*, *K_s_* |
| Time information | Year, days after sowing | Time steps, initial time, final time |
| Water flow boundary | Distance from dripper | Upper/lower boundary condition (BC); initial condition |
| Irrigation | Salinity in irrigation water, soil matric potential and irrigation water volume | Pressure head/groundwater level during different time |

Note: ML represent machine learning used in this study. *θ*_s_ and *θ*_r_ represent saturated and residual soil water contents, respectively; *α* and n denote parameters in the [soil water retention](https://www.sciencedirect.com/topics/agricultural-and-biological-sciences/soil-water-retention) function; *Ks* is the [saturated hydraulic conductivity](https://www.sciencedirect.com/topics/agricultural-and-biological-sciences/saturated-hydraulic-conductivity). Upper BC include constant pressure head, constant flux, atmospheric BC with surface layer, atmospheric BC condition with surface run off; variable pressure head, variable pressure head/flux. Lower BC include constant pressure head, constant flux, variable pressure head, variable pressure head/flux, free drainage, deep drainage, seepage face, horizontal drains.

Table S2 Main hyperparameters setting of machine learning models

| ML models | hyper_parameter |
| --- | --- |
| GBDT | max_depth=4,n_estimators=2000,random_state=2 |
| RF | n_estimators=1000,max_depth=7 |
| XGBR | max_depth=6,n_estimators=100 |

When we choose the potential ML models, a simple test including other basic ML models such as support vector machine (SVM) and multiple perceptions (MLP)was carried out. However, the preliminary results showed that their performance was significantly worse than those tree-based models (Fig. S1). Hence, we just compared those tree-based models and their ensemble integrated model in this study.


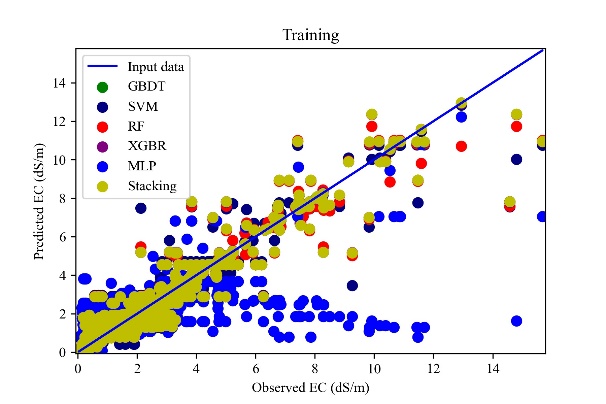

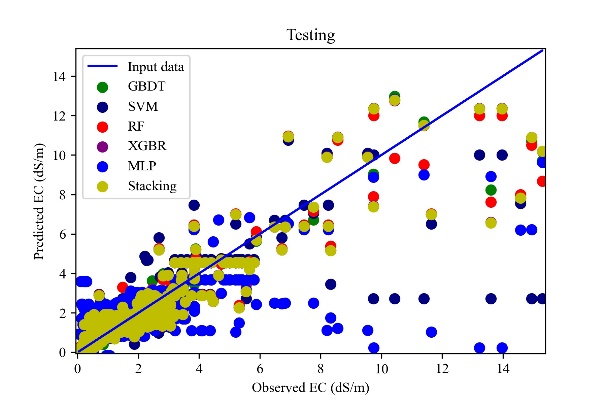


Fig. S1 Comparison of tree-based ML models (GBDT, RF, XGBR, Stacking ensemble) with SVM and MLP

$\text{ET}\text{ = }\text{I}\text{ +}\text{P}\text{±∆}\text{S-R-D}$ (1)

where I, P, ΔS, R, D represent the irrigation amount, precipitation, the change of soil water storage, surface runoff, and the downward flux below the crop root zone respectively.

**
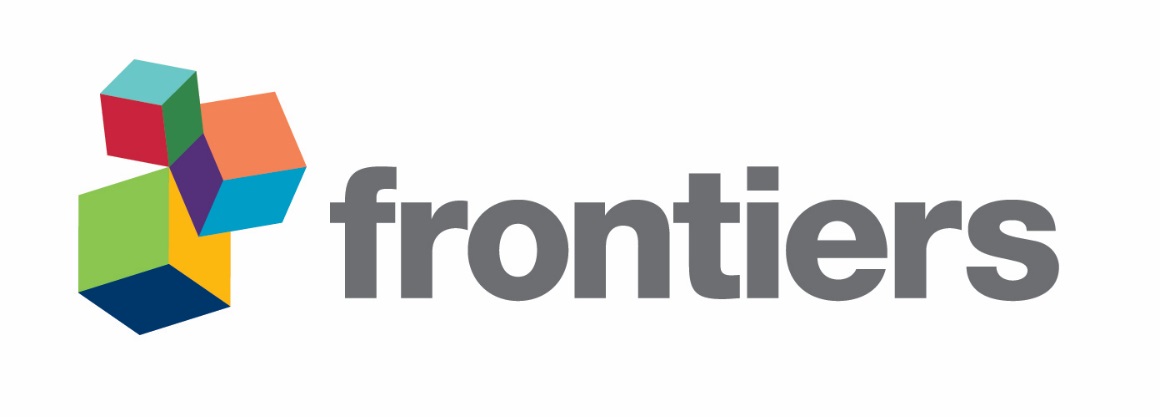
**
